# Nasolabial Ulcerated Placard Revealing a Primary Cutaneous Aggressive Epidermotropic CD8+ T-cell Lymphoma

**DOI:** 10.1155/2022/5672783

**Published:** 2022-08-09

**Authors:** J. B. Ntihebuwayo, F. Hali, A. Kerouach, H. Rachadi, S. Chiheb, F. Marnissi

**Affiliations:** ^1^Dermatology and Venereology Department, Ibn Rochd University Hospital-Casablanca, Casablanca, Morocco; ^2^Laboratory of Pathological Anatomy, Ibn Rochd University Hospital-Casablanca, Casablanca, Morocco

## Abstract

Primary cutaneous aggressive epidermotropic CD8+ T-cell lymphoma is a rare entity representing less than 1% of cutaneous lymphomas. It has an aggressive clinical manifestation with a poor prognosis. It is characterized by cytotoxic and epidermotropic CD8+ proliferation. It also expresses the TIA-1 marker. We report a new case for its display and aggressive character, diagnostic difficulty, and good therapeutic response to chemotherapy. This is a 62-year-old female patient admitted to the hospital for a nasolabial ulcerated placard evolving for two years. Clinical examination revealed submandibular lymph nodes. The specimen analysis associated with anatomoclinical manifestation was concluded for a primary cutaneous aggressive epidermotropic CD8+ T-cell lymphoma. Tumor extent assessment did not show any secondary localization. The blood tests and serology were unremarkable. The patient had benefited from a CHOEP-type multidrug therapy protocol with complete healing of the lesion after three courses of chemotherapy.

## 1. Introduction

Primary cutaneous aggressive epidermotropic CD8+ T-cell lymphoma is a rare and provisional entity according to the 2017 revised WHO classification. It represents less than 1% of all cutaneous lymphomas. It is characterized by the proliferation of epidermotropic CD8+ cytotoxic T cells with TIA-1 marker expression. It is an aggressive tumor with a poor prognosis. It affects adults, without gender predominance and without predisposing factors [1, 2]. Slowly and localized progression forms are rarely described, while diffuse forms are the most frequent [1–3]. We report an atypical case of primary cutaneous aggressive epidermotropic CD8+ T-cell lymphoma of nasal localization with a good response to chemotherapy.

## 2. Observation

**Figure 1:**
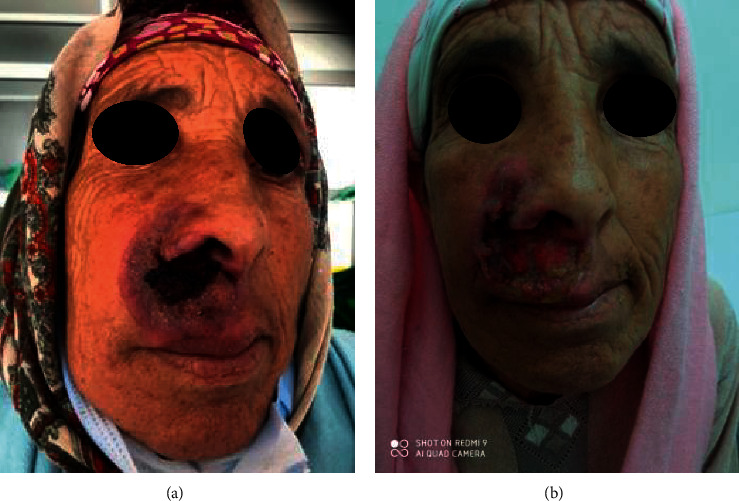
(a) Ulcerated and necrotic right nasolabial plaque with destruction of the right nasal wing on day 1 of the consultation. (b) Ulcerated and necrotic nasolabial plaque with significant loss of substance one month later after the first day of consultation.

A 62-year-old female patient was hospitalized with an erythematous and pruritic papular lesion of the right upper lip that had been evolving for two years. The lesion was infiltrated at the base with ulceration in its center and destruction of the right wing of the nose (Figure 1).

**Figure 2:**
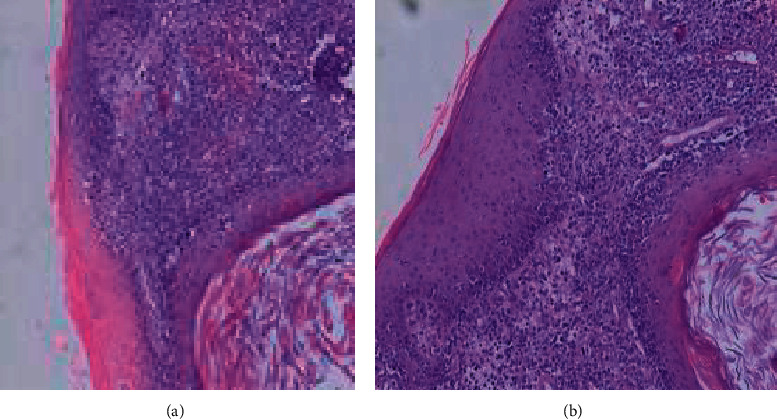
(a) Diffuse lymphoid infiltrate of the dermis and hypodermis epidermotropism, (b) diffuse lymphoid infiltrate of the dermis and hypodermis epidermotropism.

The clinical examination found two submandibular nodes with altered general condition. There was no nasal obstruction or discharge. Biopsy of the lesion showed diffuse lymphoid cell proliferation in layers occupying the entire dermis and hypodermis with epidermotropism and skin ulceration. The cells were of medium size with reduced cytoplasm and rounded or cleaved nuclei with 6 mitoses/10 HPF (Figure 2).

**Figure 3:**
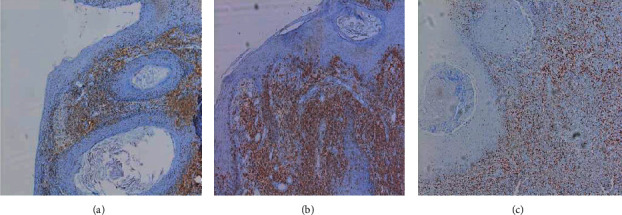
Immunohistochemistry had shown a lymphoid infiltrate expressing (a) CD3 +: positive, (b) CD8 + positive, and (c) KI67: proliferative index estimated at 50%.

Immunohistochemistry showed a lymphoid infiltrate expressing CD3, CD8, and granzyme B. The cytotoxic marker T1A1 was positive. Ki67, a proliferative index, was estimated at 50% (Figure 3). CD4, CD5, CD 20, PAX5, EBV, CD30, CD56, CD10, CD23, BCL6, and MUMI were negative.

After anatomic and clinical confrontation, the diagnosis of a primary cutaneous aggressive epidermotropic CD8+ T-cell lymphoma was retained.

The biological tests did not show any inflammatory or infectious disease, and the viral and syphilitic tests were negative (Table 1).

**Figure 4:**
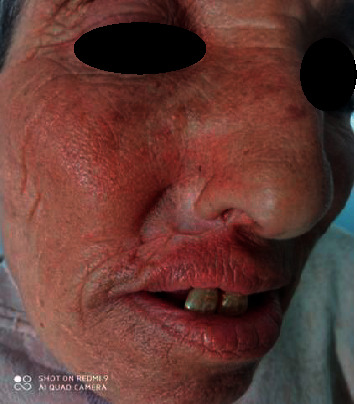
Complete healing of the lesion three months after the last chemotherapy treatment.

The cervico-cranio-facial CT scan showed a tumor process invading the right nostril and the vestibule without maxillary bone or sinus invasion. The thoraco abdominopelvic CT scan did not show any tumor localization. Aggressive multidrug therapy (CHOEP protocol) was initiated (Endoxan, doxorubicin, vincristine, onset, etoposide, and Isone). After three doses of multidrug therapy, the lesion was completely healed (Figure 4). The patient has been in complete remission for 7 months after the last chemotherapy treatment.

## 3. Discussion

The particularity of our case is its rarity, clinical presentation, and evolution but also therapeutic which lies on chemotherapy. Clinically, primary cutaneous aggressive epidermotropic CD8 + T-cell lymphoma is characterized by the sudden and generalized appearance of extensive and ulcerated papules, placards, or tumors, with or without mucosal and/or visceral involvement [1, 3].

Localized forms, especially facial, of slower evolution have been rarely reported in the literature [1, 2].

Our patient presented with a localized facial involvement with an initially slow then rapidly aggressive evolution posing a problem of differential diagnosis with a nasal-type T/NK lymphoma.

The positive diagnosis requires an anatomoclinical confrontation. Histologically, the lymphoid infiltrate is nodular or diffuse dermohypodermal with a pagetoid or lichenoid epidermotropism. The cells are small to large with irregular hyperchromatic nuclei and pleomorphic cytology. The presence of keratinocyte necrosis indicates the cytotoxic nature of the tumor cells [2, 4–6]. Immunohistochemically, the CD3+, CD8+, CD4-, CD45RA+, CD45RO−, granzyme B+, perforin+, TIA-1+, and beta-F1+ phenotype is constant. The CD56, CD2, CD7, CD5, CD15, BCL2 and *β*F1+, and MIB-1 phenotype is variable. Loss of CD2/CD5 antigens would reflect rapid progression, and CD2+/CD7—phenotype would favor indolent progression. MIB-1 shows a high proliferation index with Ki67 > 75%. The CD4, CD5, CD15, CD25, CD1a, ALK1, CD56, CD57, CD45RO, EBV/EBER, EBNA2, and LMP1 phenotypes are negative. Rare cases have been reported with the CD30 phenotype. Molecular biology shows clonal rearrangement of TCR genes and cytogenetics shows haploinsufficiency for TP53 [1, 2, 4, 6].

Our patient had a lymphoid infiltrate expressing CD3, CD8, and granzyme B with a positive TIA1 and a high proliferation index of 50%. The main differential diagnosis in our case was extranodal nasal-type T/NK cell lymphoma. It combines upper respiratory tract involvement and skin involvement with ulcerated nodules or tumors. The evolution is rapidly progressive. The phenotypes EBV and EBR and CD3+ and CD56+ are positive. It has a very poor prognosis [2, 4, 7].

Other differential diagnoses are primary cutaneous anaplastic large cell lymphoma, lymphomatoid papulosis, pagetoid T-cell lymphoma, primary cutaneous acral CD8+ T-cell lymphoma, Ketron-Goodman disease, and primary cutaneous *γδ* T-cell lymphoma [1, 4, 6, 8]. Treatment is not well codified and is based on multidrug therapy with different protocols: CHOP (Endoxan, doxorubicin, vincristine, onset, and Isone), CHOEP (Endoxan, doxorubicin, vincristine, onset, etoposide, and Isone), hyper CVAD (Endoxan, vincristine, Adriamycin, and dexamethasone). The response is unsatisfactory and usually partial [1, 9]. Partial or complete responses have been reported in the literature with electron therapy [9, 10]. Stem cell transplantation with or without chemotherapy or anti-CD 52 monoclonal antibody has been reported in the literature with partial efficacy [1, 3, 10]. Despite the poor therapeutic response reported in the literature, our patient responded well to multidrug therapy. The prognosis is unfavorable with an aggressive clinical course. Survival at 5 years is estimated between 18 and 32% with a median survival of 12–32 months. Diagnosis at the tumor lesion stage is associated with a poor prognosis [1, 3, 8, 10, 11].

## 4. Conclusion

Primary cutaneous aggressive epidermotropic CD8+ T-cell lymphoma is a rare entity with a poor prognosis. Its clinical manifestation and history are unusual. The presentation and clinical history of our patient are unusual and constitute a diagnostic challenge, hence the interest of anatomical and clinical confrontation to confirm the diagnosis. Despite the poor therapeutic response reported in the literature, our patient responded well to chemotherapy, hence the interest of monitoring. Early diagnosis and management could improve patient survival.

## Figures and Tables

**Table 1:** Biological workup performed.

Realized balance sheet	Value recovered	Usual value
Hb	11, 2	12–16 g/dl
Lymphocytes	1170	1000–5000 cells/*µ*L
VS	21	<20 mm in young women
CRP	1, 7	1–4 mg/L
LDH	174	125–220 UI/L
ASAT	17	5–34 UI/L
ALAT	13	0–55 UI/L
Urea	0, 27	0, 13–0, 43 g/L
Creatinine	6, 1	5, 7–11, 1 mg/L
TPHA	Negative	
VDRL	Negative	
EBV	Negative	
Anti-HIV 1-2 combined	Negative	
Anti HTLV 1 antibodies	Negative	

Hb: hemoglobin, ASAT: aspartate aminotransferase, ALAT: alanine aminotransferase, LDH: lactate dehydrogenase, CRP: C-reactive protein, VS: sedimentation rate, TPHA: *Treponema pallidum* hemagglutination assay, VDRL: venereal disease research laboratory, anti-HIV 1-2 combined: human immunodeficiency virus, EBV: Epstein–Barr virus, HTLV-1: human T-lymphotropic virus.

## Data Availability

The data that support the findings of this study are available from the corresponding author upon reasonable request.
